# Detection of calcified plaques: comparison between coronary CT angiography and thin-slice non-contrast CT with deep learning-aided image registration

**DOI:** 10.1007/s00330-026-12501-y

**Published:** 2026-04-20

**Authors:** Kenrick Schulze, Federico Biavati, Bernhard Föllmer, Sotirios Tsogias, Surenjav Chimed, Hanna Balogh, Norbert Nagy, Ferhat Yavuz, Anne-Marieke Stantien, Renad-Heyam Abdelrahman, Steffen Lukas, Maria Bosserdt, Marc Kachelrieß, Wojciech Samek, Marc Dewey

**Affiliations:** 1 Department of Radiology, Charité–Universitätsmedizin Berlin, corporate member of Freie Universität Berlin, Humboldt-Universität zu Berlin, Berlin, Germany; 2 Inria, Université Côte d’Azur, Epione team, Sophia Antipolis, France; 3 Institute of Medical Sciences, National University of Medical Sciences, Ulaanbaatar, Mongolia; 4 Coronary Care Unit, Shastin’s Third State Central Hospital, Ulaanbaatar, Mongolia; 5 Department of Radiology, Medical Imaging Centre, Semmelweis University, Budapest, Hungary; 6 German Cancer Research Center (DKFZ), Heidelberg, Germany; 7 Heidelberg University, Heidelberg, Germany; 8 Department of Artificial Intelligence, Fraunhofer Heinrich Hertz Institute, Berlin, Germany; 9 Department of Electrical Engineering and Computer Science, Technical University of Berlin, Berlin, Germany; 10 BIFOLD (Berlin Institute for the Foundations of Learning and Data), Berlin, Germany; 11 BIH (Berlin Institute of Health), DHZC (German Heart Center of the Charité) and DZHK (German Center for Cardiovascular Research), Berlin, Germany

**Keywords:** Coronary computed tomography angiography, Coronary artery calcification, Image registration, Artificial intelligence

## Abstract

**Objectives:**

To investigate whether coronary CT angiography (CCTA) misses calcified plaques detected by thin-slice non-contrast CT (NCCT).

**Materials and methods:**

This study included patients from two sites in the DISCHARGE trial for whom both 0.5 mm thin-slice NCCT and CCTA were available. Plaques on CCTA were defined as missed if they showed no spatial overlap with NCCT-detected plaques after deep learning-aided co-registration. Comparisons of plaque volume, density, and local coronary luminal attenuation between plaques missed and those detected by CCTA were performed using the Mann–Whitney U-test. In addition, the presence of these plaques on standard calcium scoring CT was assessed. Interobserver agreement was assessed using the intraclass correlation coefficient and Bland‒Altman analysis.

**Results:**

This study included 45 patients (40% female, mean age 62 ± 11 years), in whom CCTA missed 37.6% of calcified plaques detected by NCCT (121/322). Missing calcified plaques on CCTA misclassified 8.9% of patients (4/45) as having no plaques. Compared with detected plaques, plaques missed by CCTA were both significantly smaller in volume (3.0 mm³ [IQR, 1.5–4.9] vs. 9.2 mm³ [IQR, 4.3–21.9], *p* < 0.001) and had lower density (212.7 HU [IQR, 174.5–242.4] vs. 292.7 HU [IQR, 243.2–361.3], *p* < 0.001). Only 44.0% of plaques (53/121) missed by CCTA were detected by standard calcium scoring CT. Interobserver analysis demonstrated excellent agreement for calcified plaque volume on CCTA (ICC = 0.91) and NCCT (ICC = 0.98).

**Conclusion:**

CCTA missed more than one-third of coronary calcified plaques that are identifiable on co-registered thin-slice NCCT, which suggests an underutilized role of thin-slice NCCT in clinical practice.

**Key Points:**

***Question*** Accurate detection of all coronary plaques is crucial for risk stratification. CCTA misses calcified plaques, which are detectable by thin-slice non-contrast CT (NCCT).

***Findings*** CCTA misses over one-third of calcified plaques, nearly half of which are also missed by calcium scoring CT. NCCT detected these calcified plaques.

***Clinical relevance*** Deep learning-aided registration enables multimodal CCTA–NCCT assessment, improving detection of calcified plaques overlooked by CCTA alone and providing more accurate plaque burden quantification that may support better clinical decision-making, which should be investigated in future studies.

**Graphical Abstract:**

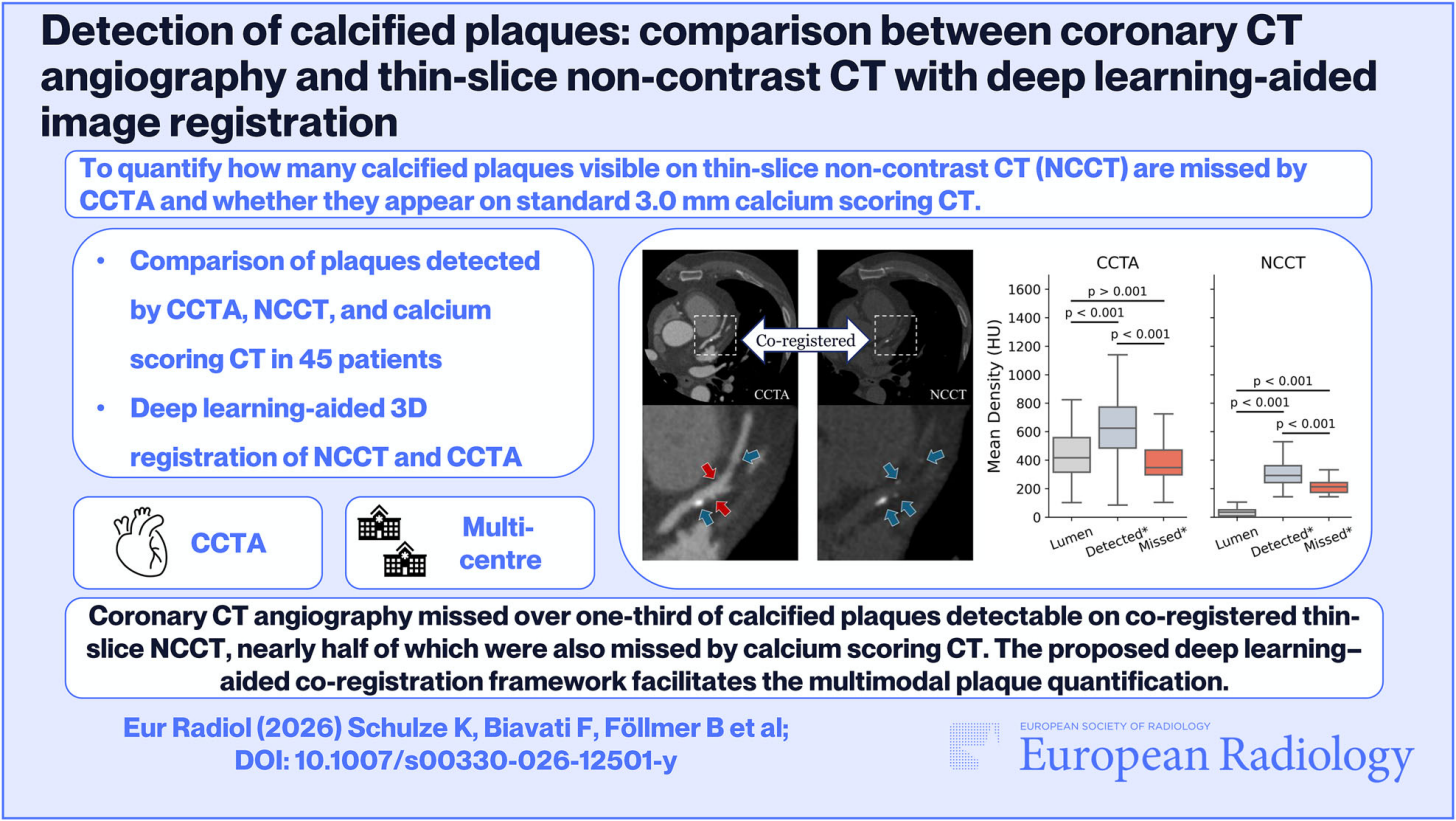

## Introduction

Non-contrast calcium scoring CT is routinely performed alongside coronary computed tomography angiography (CCTA) for diagnosing coronary artery disease (CAD). The coronary calcium score observed on calcium scoring CT quantifies the calcium burden on the arteries at the patient level and has been shown to be an independent marker for subsequent coronary events [1]. Luminal stenosis assessment and plaque quantification are performed on CCTA due to the absence of contrast agents, as well as the lower signal-to-noise ratio and limited spatial resolution of 3.0 mm calcium scoring CT reconstructions [2, 3]. Accurate detection and quantification of all coronary plaques is critical for optimal patient care. Even small, calcified plaques are indicative of subclinical atherosclerosis and are associated with increased long-term cardiovascular risk, whereas the absence of coronary artery calcium is associated with a low prevalence of obstructive CAD (< 5%) [4].

However, the presence of calcified plaques may be underestimated by radiologists in clinical practice when using CCTA alone due to masking effects from intraluminal iodine attenuation and blooming artifacts, particularly when assessing low-to-intermediate-risk patients with low plaque burden [5]. Using calcium scoring CT together with CCTA has been shown to improve CAD diagnosis compared to CCTA alone [6], but few studies have evaluated its benefit at the plaque-level.

Baliyan et al [5] reported that up to 41% of calcified plaques detected on calcium scoring CT were missed on CCTA, particularly when low-voltage acquisition protocols were used. Their study was performed on standard 3.0 mm reconstructions, which can obscure small plaques due to partial volume effects [7, 8]. Thin-slice 0.5 mm non-contrast CT (NCCT) reconstructions provide this additional spatial resolution compared to conventional calcium scoring [9].

Therefore, in this study, we investigated the added value of thin-slice NCCT in detecting calcified coronary artery plaques compared to using CCTA or calcium scoring CT alone. For this purpose, we developed a deep learning-aided registration tool that aligns plaques found by CCTA and thin-slice NCCT to enable multimodal plaque quantification.

## Materials and methods

This is an exploratory subgroup analysis of the DISCHARGE (Diagnostic Imaging Strategies for Patients with Stable Chest Pain and Intermediate Risk of Coronary Artery Disease) trial (NCT02400229) which was approved by the ethics committee at Charité–Universitätsmedizin Berlin as the coordinating center (EA1/294/13), by the German Federal Office for Radiation Protection, and by local or national ethics committees [10]. All patients provided written informed consent.

### Patient cohort

This prospective analysis (Statistical Analysis Plan: Table 19 [11]) included patients from the DISCHARGE trial, a prospective, pragmatic, multicenter randomized study conducted at 26 centers across 16 European countries. Patients were enrolled between October 3, 2015, and April 12, 2019. The DISCHARGE trial compared the effectiveness and safety of CCTA with invasive coronary angiography (ICA) in patients with a low-to-intermediate pretest probability (10–60%) of obstructive CAD. This analysis included patients for whom thin-slice (0.5 mm) NCCT and CCTA were available. Samples were excluded if the presence of severe image artefacts (e.g., severe motion artefacts) made the examination with either modality infeasible.

### Image acquisition

The CT images included in this study were acquired with prospective electrocardiographic (ECG) triggering on a 320-row CT scanner (Aquilion ONE, Canon Medical Systems). Acquisition parameters included a small field of view, 120 kV, and a tube current tailored to the patients’ size. Non-contrast images were acquired before CCTA during the same examination. According to protocol, calcium scoring CT images were reconstructed with a slice thickness and spacing of 3.0 mm. For a subset of patients, thin-slice reconstructions have been available at two centers with a slice thickness and spacing of 0.5 mm.

### Image analysis

The diagnostic accuracy of manually segmented coronary calcified plaques was semi-automatically assessed for thin-slice NCCT and CCTA. For the detection of calcified plaques in the thin-slice NCCT, we set a minimal attenuation threshold of 130 Hounsfield units (HU), in line with the definition of Agatston et al [12] for calcium scoring. Thin-slice NCCT reconstructions have a lower signal-to-noise ratio than standard 3.0 mm reconstructions. Thus, choosing a threshold of 130 HU can result in false positive findings. Therefore, we additionally required that the size of calcified plaques is visually larger and denser than image noise on the same axial slice. While very small calcifications near the noise level may be excluded, this reduces the risk of false positive findings.

Additionally, coronary calcium scoring was performed on standard 3.0 mm calcium scoring CT reconstructions, when available, using the Agatston method. Calcified plaques were segmented by an independent observer with at least 1 year of experience in calcium scoring. The segmentation of calcified plaques in thin-slice NCCT and calcium scoring CT was performed using 3D Slicer V 5.4.2 [13].

Quantitative atherosclerotic coronary plaque analysis in CCTA was performed using the semi-automatic software AutoPlaque (Version 3.0, Cedars-Sinai Medical Center [14]) by two resident physicians with at least 1 year of training in cardiac imaging, who were blinded to the non-contrast CT images. Using a semi-automated method, centerlines, vessel walls, and lumen boundaries were extracted for the main coronary arteries and branches, with manual correction applied as needed. A reference of normal blood pool attenuation was defined by placing a region of interest in the proximal aorta at the level of the ostium of the left and right coronary arteries to define the normal reference blood pool attenuation. Proximal and distal markers covering coronary artery segments with atherosclerotic plaques were placed manually. For each delineated plaque, its composition was automatically quantified based on adaptive scan-specific thresholds.

### Image registration

**Fig. 1:**
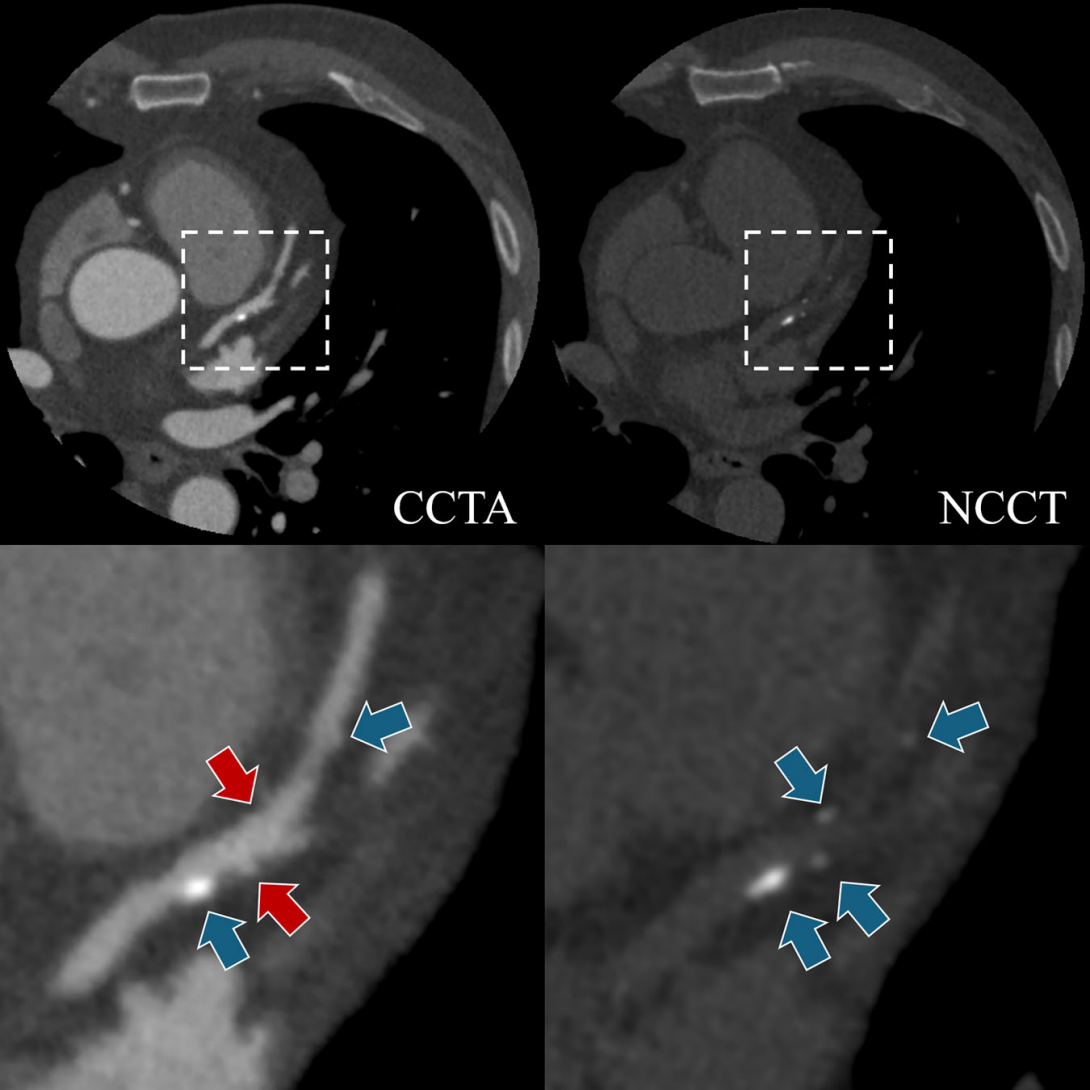
62-year-old male patient with calcified plaques missed by coronary CT angiography (CCTA) but detected by thin-slice non-contrast CT (NCCT). Thin-slice NCCT detected four distinct calcified plaques in the depicted segment of the left anterior descending artery (LAD). In CCTA, two of these plaques were missed (red arrows)

Calcified plaque matching between thin-slice NCCT and CCTA was performed using a fully automatic approach. After co-registration of the thin-slice NCCT and CCTA scans, plaques were classified as missed when no spatial overlap was detected between the two modalities (Fig. 1).

**Fig. 2:**
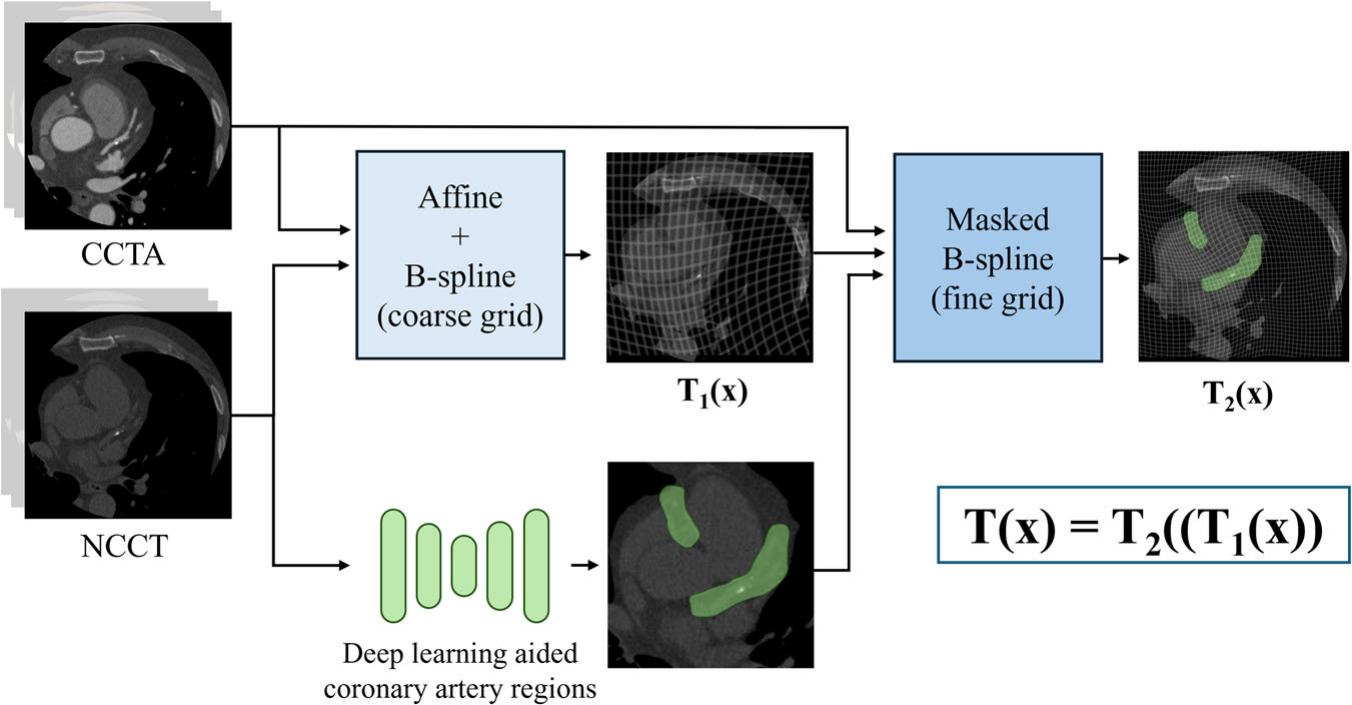
Deep learning-aided 2-stage 3D registration framework $T\left(x\right)$ of the 3D volume x. Thin-slice non-contrast CT (NCCT) 3D volumes are warped, gradually aligning with coronary CT angiography (CCTA). The first registration stage accounts for global differences. The resulting transformation ${T}_{1}\left(x\right)$ serves as the input for the second stage ${T}_{2}(x)$, which is optimized over a fine grid of control points within the coronary artery regions (CAR) extracted from NCCT using a deep learning convolutional network described in [16]

The registration of cardiac structures is challenging due to both local respiratory-induced heart motion and global patient movements, particularly when registering thin-slice NCCT and CCTA. The intensity profiles of tissues such as the myocardium, vessels, and fatty tissue can differ significantly between these two modalities, posing significant challenges to conventional registration algorithms. We developed a 2-stage non-rigid multiresolution registration algorithm with a scaling pyramid schedule using the elastix toolkit [15]. In the first stage, a composite transform ${T}_{1}$(x) of an affine and a B-spline transformation was optimized to align both sequences along a coarse grid of control points (Fig. 2). This global registration is driven by strong boundaries such as ribs and spinal columns and thus fails to register the coronary arteries precisely. We therefore performed a second B-spline transformation ${T}_{2}$(x), which was optimized by sampling points exclusively within regions enclosing the coronary arteries in the thin-slice NCCT images (Fig. 2).

The coronary artery regions were generated using a multi-task deep learning network for the segmentation of calcified plaques in calcium scoring CT, as proposed by Föllmer et al [16]. Here, the segmentation task is guided by the auxiliary task of predicting coronary artery regions. We applied the trained model to thin-slice NCCT, extracted only the coronary artery regions, and subsequently dilated them to ensure inclusion of artery regions for both modalities after the first registration stage (Supplementary Fig. 1). The registration tool enabled the multimodal assessment of plaques (Supplementary Fig. 3) by providing only 3D volumes of thin-slice NCCT and CCTA without any preprocessing required. Further details about the 2-stage registration framework are described in Supplementary Table 2 and Supplementary Fig. 2.

### Matching of calcified plaques

For the semi-automated matching of calcified plaques, the segmentations of calcified plaques in the CCTA were dilated using a 1-connectivity kernel with two iterations to avoid falsely considering plaques as missed due to minor misalignments after registration. Plaques were defined as missing if there was no overlap after registration and dilation between modalities. Dilation was performed only in CCTA. Hereby, thin-slice NCCT was used as the technical reference modality, as no external gold standard, such as IVUS, was available. To minimize the risk of spurious calcified plaque segmentations caused by noise in thin-slice NCCT, discrepancies between thin-slice NCCT and CCTA segmentations were manually validated. Segmentations in the thin-slice NCCT that were identified as potential calcified plaques were discarded as false positives if no corresponding morphology was detected within the coronary arteries on the CCTA.

### Statistical analysis

Quantitative data is expressed as mean and standard deviation, or median and interquartile range (IQR), depending on the distribution of the data. The Mann–Whitney U-test was used to compare plaque volume and attenuation values between missed plaques and all detected plaques, while the χ² (chi-square) test was used to compare the number of detected plaques between thin-slice NCCT and calcium scoring CT. Additionally, the attenuation values of the coronary lumen were compared with those of the coronary plaques. A *p*-value of less than 0.05 was considered statistically significant. Interobserver agreement was assessed using the intraclass correlation coefficient (ICC) with 95% confidence intervals (CI). Following the methods of Koo and Li [17], we interpreted ICC values of less than 0.5, between 0.5–0.75, between 0.75–0.9, and greater than 0.9 as poor, moderate, good, and excellent, respectively. Agreement between observers for the number of detected plaques and plaque volume was additionally assessed at both the vessel and patient levels using Bland–Altman plots. Statistical analysis was performed using Python 3.11.

## Results

### Study population

Within the CT arm of the DISCHARGE trial, 0.5 mm thin-slice NCCT scans were available for 50 patients. After further exclusion of non-diagnostic images with severe artefacts, this study was performed on 45 patients (40% female), with a mean age of 62.0 years ± 11.3, who were recruited at two different sites. Importantly, images with artefacts that still allowed for the quantification of plaques were not excluded from the analysis. Patient characteristics are summarized in Table 1, with comparison to the remaining study cohort of the two centers shown in Supplementary Table 1.

**Table 1:** Baseline characteristics of patients

	Cohort (*N* = 45)
Patient characteristics	
Women	18/45 (40.0)
Age (years)	62.0 ± 11.3
Body mass index (kg/m^2^)	27.6 ± 5.6
Risk factors	
Hypertension	33/45 (73.3)
Diabetes	7/45 (15.6)
Hyperlipidemia	25/45 (55.6)
Smoking (current)	10/45 (22.2)

All values are expressed as the mean ± standard deviation or number (%)

### Patient-level comparison

**Fig. 3:**
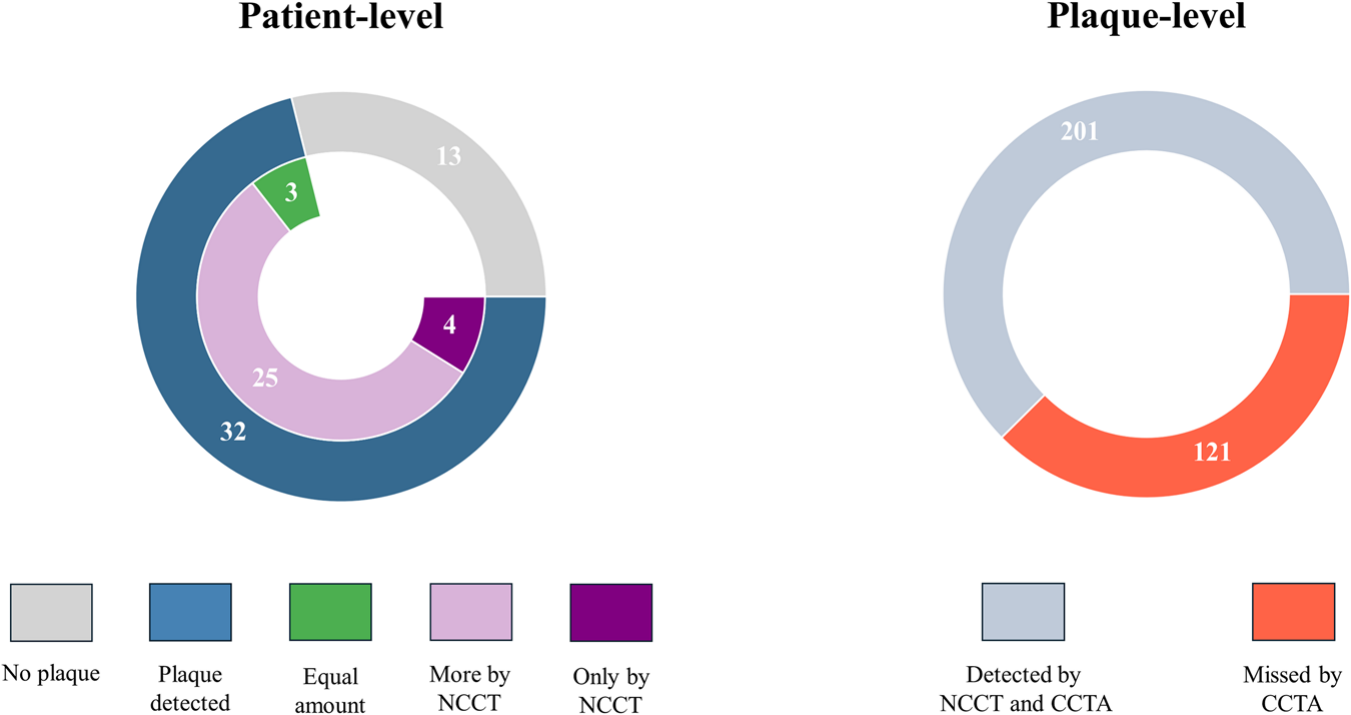
Number of calcified plaques detected by thin-slice non-contrast CT (NCCT) and coronary CT angiography (CCTA), shown by patient count and number of plaques detected. Only for a fraction of patients, CCTA and thin-slice NCCT detected the same plaques per patient (“Equal amount”). For most patients, thin-slice NCCT detected more calcified plaques than CCTA (“More by NCCT”). In four patients, NCCT identified calcified plaques that were completely missed by CCTA (“Only by NCCT”)

At the patient level, thin-slice NCCT identified calcified coronary plaque in 71.1% of patients (32/45). Using CCTA alone, all calcified plaques were correctly identified in only 35.6% of patients (16/45) (Fig. 3). Among the 32 patients with at least one calcified plaque on thin-slice NCCT, CCTA missed at least one plaque in 90.6% of patients (29/32). Furthermore, if CCTA alone had been used for plaque detection, 8.9% of patients (4/45) would have been classified as having no coronary plaque despite the presence of calcification on thin-slice NCCT.

### Plaque-level comparison

A total of 322 calcified plaques were identified by thin-slice NCCT in the 45 included data sets. Of these 322 plaques, 37.6% (121/322) were missed by CCTA alone (Fig. 3).

**Fig. 4:**
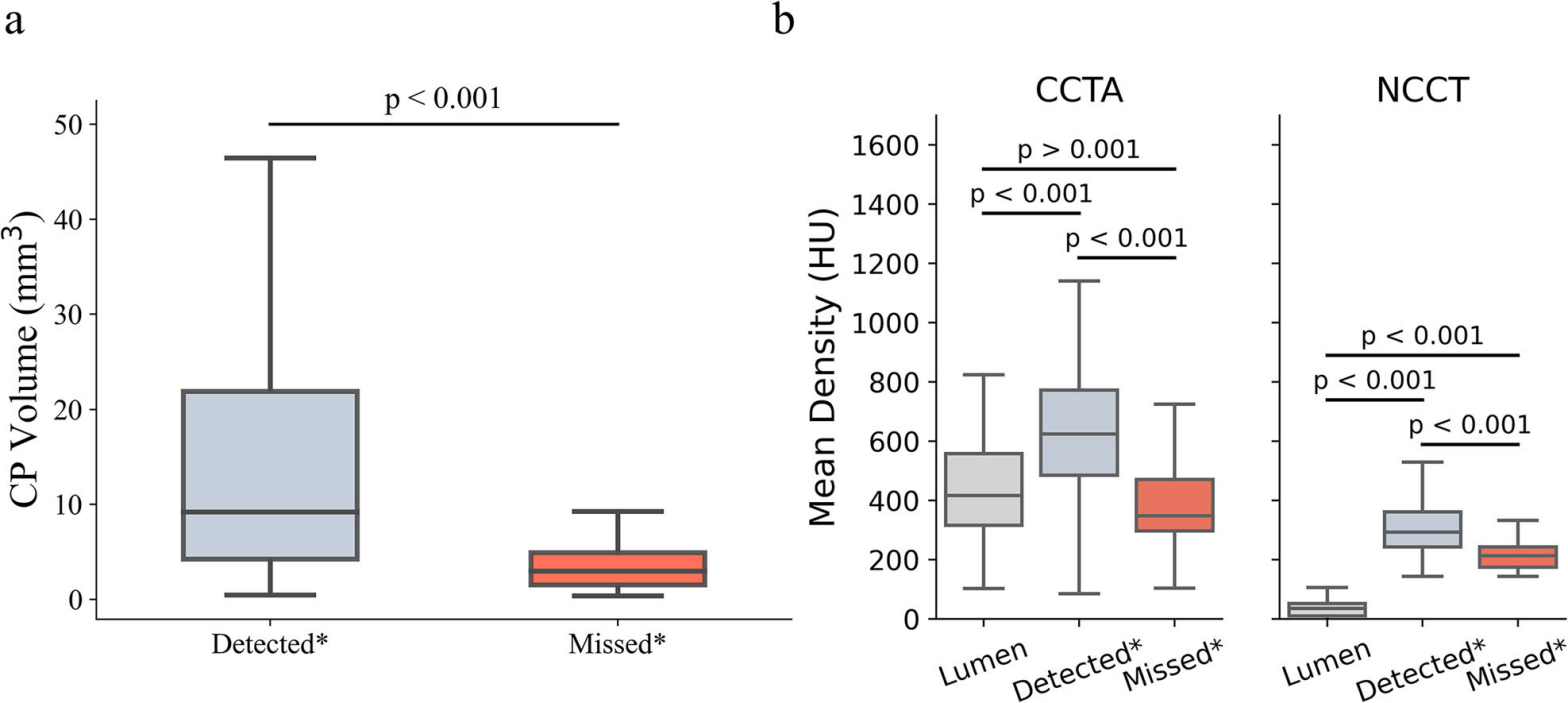
Quantification of calcified plaques grouped by detected and missed by coronary CT angiography (CCTA) alone. The distribution of calcified plaques missed by CCTA alone was smaller in volume compared to all detected plaques (**a**). Grouped box plot demonstrating the distribution of Hounsfield units (HU) of mean aortic attenuation, detected plaques, and plaques missed by CCTA alone, quantified on CCTA and thin-slice non-contrast CT (NCCT), respectively (**b**). “*” refers to detected and missed by CCTA alone

Compared with all detected plaques, coronary plaques that were missed by CCTA alone were significantly smaller in volume (3.0 mm³ [IQR, 1.5–4.9 mm³] vs. 9.2 mm³ [IQR, 4.3–21.9 mm³], *p* < 0.001) (Fig. 4a) and exhibited significantly lower attenuation (212.7 HU [IQR, 174.5–242.4 HU] vs. 292.7 HU [IQR, 243.2–361.3 HU], *p* < 0.001). In contrast to thin-slice NCCT, the density of plaques detected on CCTA was close to the luminal density, impeding the correct detection of all calcified plaques (Fig. 4b).

Furthermore, there was no significant difference in attenuation between plaques missed by CCTA and those missed by luminal density on CCTA (347.7 HU [IQR, 297.4–471.1 HU vs. 417.0 HU [IQR, 316.1–558.2 HU], *p* = 0.08). Conversely, when assessed via thin-slice NCCT, calcified plaques demonstrated significantly greater attenuation than the lumen (212.7 HU [IQR, 174.5–242.4 HU] vs. 35.0 HU [IQR, 9.5–51.9 HU], *p* < 0.001), which facilitated simple visual identification by the readers.

### Comparison with calcium scoring CT

**Fig. 5:**
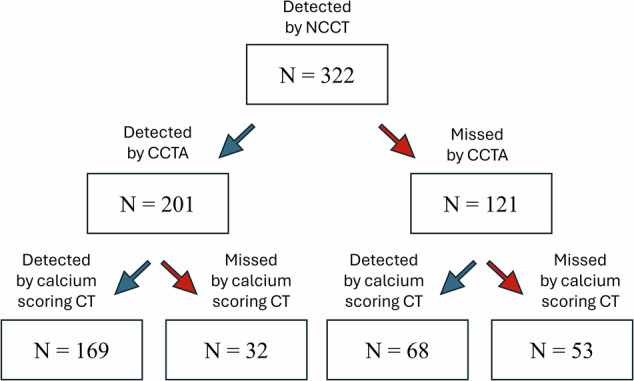
Flowchart illustrating the distribution of calcified plaques detected and missed by coronary CT angiography (CCTA) and calcium scoring CT, using thin-slice non-contrast CT (NCCT) as reference

CCTA missed a greater proportion of calcified plaques than calcium scoring CT (37.6% [121/322] vs. 26.4% [85/322]). Among plaques missed by CCTA, 44% (53/121) were also missed by calcium scoring CT, whereas calcium scoring CT missed only 15% (32/201) of plaques detected by CCTA (Fig. 5), resulting in a significantly different distribution of plaque detection between thin-slice NCCT and calcium scoring CT relative to CCTA (χ² = 4.428, *p* < 0.05).

### Interobserver variability analysis

**Fig. 6:**
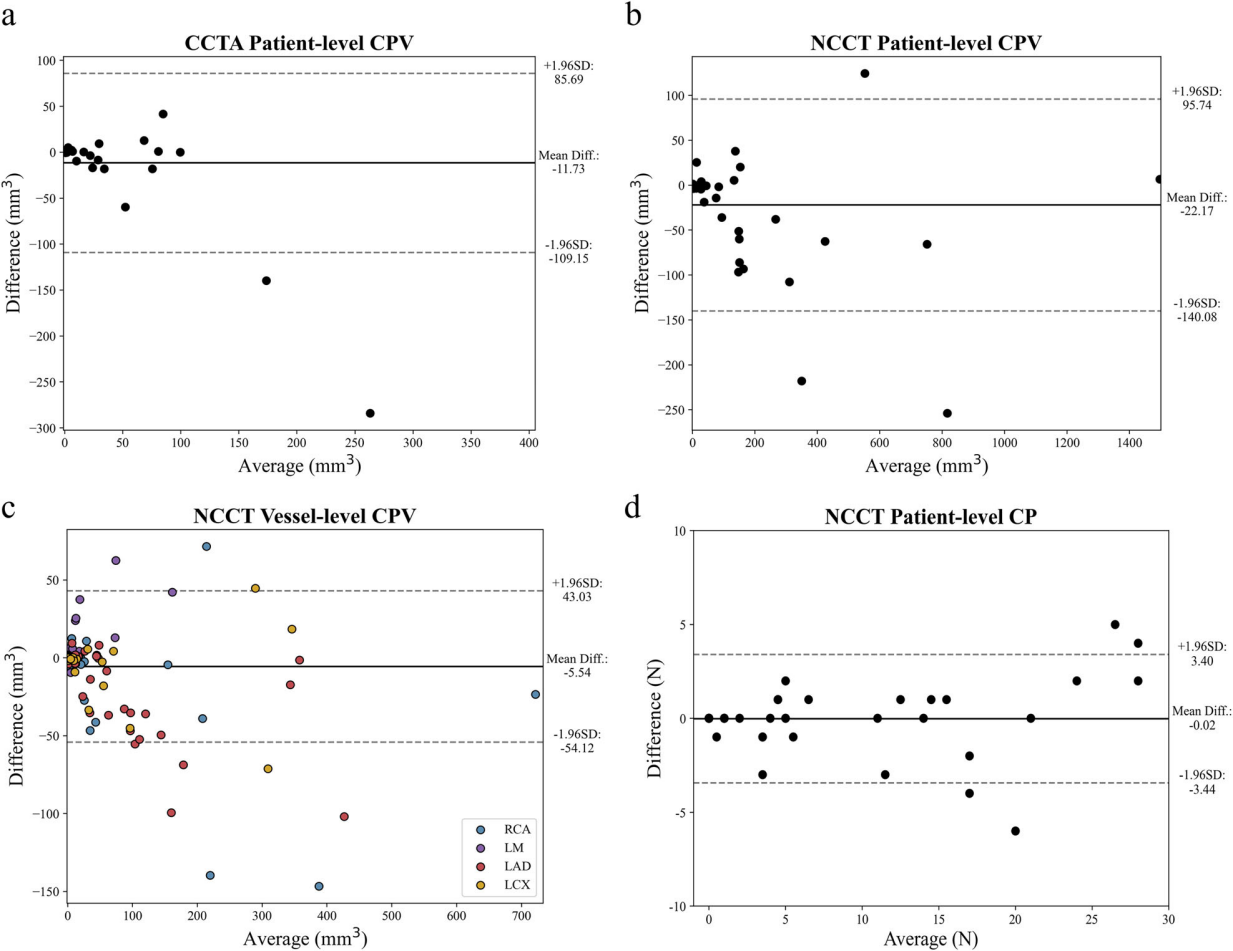
Bland–Altman analysis comparing calcified plaque detection on coronary CT angiography (CCTA) at patient level (**a**), thin-slice non-contrast CT (NCCT) at patient level (**b**), thin-slice NCCT at vessel level (**c**), and thin-slice NCCT for the number of detected plaques on patient level (**d**)

The interobserver variability was assessed using independent segmentations performed by two observers per imaging modality, with a different pair of observers for non-contrast and contrast-enhanced images. Despite the limited signal-to-noise ratio of thin-slice NCCT reconstructions, we observed an excellent ICC of 0.97 (95% CI: 0.95–0.99) for the number of detected calcified plaques per patient. We also observed an excellent ICC of 0.98 (95% CI: 0.95–0.99) and 0.91 (95% CI: 0.83–0.95) for calcified plaque volume at the patient level for thin-slice NCCT and CCTA, respectively. Moreover, we did not observe differences in interobserver agreement between vessels (Fig. 6). The strongest differences in calcified plaque volume between the two observers when evaluating thin-slice NCCT arose from differences in the perceived extent of blooming (Supplementary Fig. 4). Bland‒Altman analysis further illustrates the second observers’ tendency to delineate plaques with broader outlines, while the number of detected plaques per patient is consistent with a mean difference of 0.02 (Fig. 6d).

## Discussion

In this study, we investigated the added value of thin-slice NCCT for detecting calcified coronary artery plaques compared with CCTA alone. We developed a 2-stage registration workflow to align 3D thin-slice NCCT volumes with CCTA for multimodal coronary plaque quantification. In summary, CCTA missed 37.6% of all plaques detected by thin-slice NCCT. These plaques were smaller in volume and had an attenuation similar to that of the coronary lumen when compared to all detected plaques.

Our findings align with the results of Baliyan et al [5], who demonstrated that calcium scoring CT has additional value for the detection of calcified plaques. Accurate plaque detection is crucial for optimal risk assessment, as different levels of plaque burden affect patient management. The absence of calcified plaques has been linked in several studies to a reduced risk of future cardiac events. The randomized controlled trials DISCHARGE, SCOT-HEART, and PROMISE linked a CAC score of 0 to a strongly reduced rate of obstructive CAD over multiple years of follow-up [11, 18, 19]. On the other hand, large, calcified plaque burdens have been linked to plaque stability, which reduces the risk of plaque rupture [20]. However, our study demonstrated that small plaques were more frequently missed on CCTA. Multiple studies have indicated that small plaques in particular carry a high risk of cardiovascular events. For instance, increased incidence of spotty calcifications (< 3.0 mm) has been associated with a higher risk of plaque rupture [21] and acute coronary syndrome [22]. Spotty calcifications are high-risk coronary plaque features that have been shown to be relevant for predicting outcomes [23, 24]. Furthermore, plaque density plays an important role in risk stratification. Several studies identified plaque density to be inversely correlated with CVD [25]. Our results demonstrated that plaques missed by CCTA exhibited statistically significantly lower attenuation values and smaller volume compared to all detected calcifications. Further outcome-driven studies are required to determine whether small amounts of coronary calcification detected exclusively on thin-slice NCCT images should influence clinical decision-making. We are currently extending this work within the DISCHARGE [11]—Extend 10-year follow-up (funded by DFG) and IMPRO (G-BA: 01NVF24302) studies, which will allow us to compare thin-slice NCCT with CCTA in several thousand patients, where initial results are expected in 2030.

Our study indicates an added value of thin-slice NCCT in detecting calcified plaques compared to CCTA alone. While some recent studies have reported major adverse cardiovascular events (MACE) in patients without apparent calcified plaques, particularly younger individuals [18], it is important to consider the imaging modalities used in those studies. Despite advances in CT scanner technology and reconstruction methods, these studies relied on CCTA or calcium scoring CT to quantify plaques. This may falsely underestimate the patient’s true calcium burden, which can be more accurately assessed with thin-slice NCCT [9]. Van der Bijl et al [7] reported at least one calcification on thin-slice reconstruction in 21% of patients with a conventional calcium score of 0 (*N* = 100). Urabe et al [8] compared the prevalence of non-calcified plaques in a subgroup of patients (*N* = 132) using both calcium scoring CT and thin-slice NCCT. They found a significantly higher prevalence of plaques with non-calcified components (83% vs. 14%; *p* < 0.001) in patients where calcified plaques were detectable only on thin-slice NCCT but not on conventional calcium scoring CT. These findings highlight the need for more sensitive imaging techniques to more accurately assess subclinical atherosclerotic burden beyond conventional calcium scoring by combining thin-slice NCCT and CCTA. The improved detection rate with thin-slice reconstructions may justify the screening of low-risk individuals who may not benefit from extensive CCTA examinations. This effect is likely to be further amplified by the widespread implementation of photon-counting CT, which enables even more detailed characterization of coronary plaques [26].

Artificial intelligence has a tremendous impact on a wide range of biomedical image applications. Several models have been developed for plaque evaluation in coronary arteries on CCTA, enabling rapid detection and quantification of plaque phenotypes [27–30]. Considering the incremental value of thin-slice NCCT for detecting calcified plaques, we conjecture that a multimodal deep learning framework for plaque evaluation would benefit from simultaneously extracting features from the adjunct analysis of thin-slice NCCT and CCTA. This can be achieved by integrating uncertainties from each modality’s model into a weighted decision-making process [31]. As a first step, the proposed registration method aligns both sequences, a crucial step for accurate evaluation of multimodal plaque quantification.

Our study has several limitations that must be acknowledged. This retrospective observational study included a rather small subset of 45 patients from two centers within the DISCHARGE trial for whom thin-slice non-contrast reconstructions were available. Second, we did not assess the prognostic relevance of calcifications missed by CCTA alone. Given that the presence of any coronary plaque is a sign of vascular damage and a risk factor for MACE, plaques missed by CCTA alone may warrant early intervention to address the underlying pathophysiology of atherosclerosis, particularly in young adults, which must be evaluated in randomized studies. Furthermore, this study was conducted using a tube voltage of 120 kV. Even though the ability to distinguish iodine from calcium is limited by their similar spectral attenuation characteristics and thus expected to persist also at lower kV settings, future studies should be done to validate our results at lower kV settings and using non-iodine-based contrast media [32]. Lastly, thin-slice NCCT images were used as a technical reference modality and should be validated using invasive gold standards in future studies. Despite these limitations, our findings provide valuable insights to potentially advancing conventional risk categorization, such as the Agatston method, tailored to thin-slice NCCT reconstructions.

## Conclusion

Our study found CCTA to underestimate the coronary calcification burden, missing more than one-third of plaques detectable by co-registered thin-slice NCCT. This diagnostic gap warrants further investigation, as CCTA alone could potentially overlook certain patients who are at increased risk for future cardiovascular events. The combined multimodal approach may enhance diagnostic capabilities and might improve accurate coronary plaque quantification and effective patient risk stratification.

## Supplementary information

ELECTRONIC SUPPLEMENTARY MATERIAL

## Data Availability

The deep learning-aided registration framework will be made available upon reasonable request. The data is not publicly available due to privacy and ethical restrictions.

## Abbreviations

CAD: Coronary artery disease
CCTA: Coronary CT angiography
HU: Hounsfield units
ICC: Intraclass correlation coefficient
IQR: Interquartile range
MACE: Major adverse cardiovascular events
NCCT: Non-contrast CT
