# Supplementary material for: Detection of calcified plaques: comparison between coronary CT angiography and thin-slice non-contrast CT with deep learning-aided image registration
Source: Eur Radiol. 2026 Apr 20;36(8):6636–46. doi: 10.1007/s00330-026-12501-y (PMC13342326; doi:10.1007/s00330-026-12501-y)
Supplement: Supplementary file 1 — ELECTRONIC SUPPLEMENTARY MATERIAL [file 330_2026_12501_MOESM1_ESM.pdf]

Detection of Calcified Plaques: Comparison Between Coronary CT Angiography and Thin-Slice Non-Contrast CT with Deep Learning-aided Image Registration

ELECTRONIC SUPPLEMENTARY MATERIAL

Eur Radiol (2026) Schulze K, Biavati F, Föllmer B et al.

Table 1: Baseline characteristics of patients from the two DISCHARGE centers included in this analysis. “Included” refers to patients for whom both thin-slice non-contrast CT and CCTA were available, whereas “excluded” comprises patients in the CT group of the DISCHARGE trial who did not have thin-slice non-contrast CT available.

	**Included (N=45)**	**Excluded (N=232)**	**P-value**
Patient characteristics			
Women	18/45 (40.0)	114/232 (49.1)	0.33
Age (years)	62.0 ± 11.3	60.9 ± 10.2	0.36
Body mass index (kg/m2)	27.6 ± 5.6	28.3 ± 5.5	0.41
Risk factors			
Hypertension	33/45 (73.3)	130/232 (56.0)	0.09
Diabetes	7/45 (15.6)	29/232 (12.5)	0.75
Hyperlipidemia	25/45 (55.6)	111/232 (47.8)	0.43
Smoking (current)	10/45 (22.2)	48/232 (20.6)	0.50

Note: All values are expressed as the mean ± standard deviation or number (%) The χ^2 test (or Fisher exact test for small data sets) was used for categorical variables Mann-Whitney *U* test was used for continuous variables.

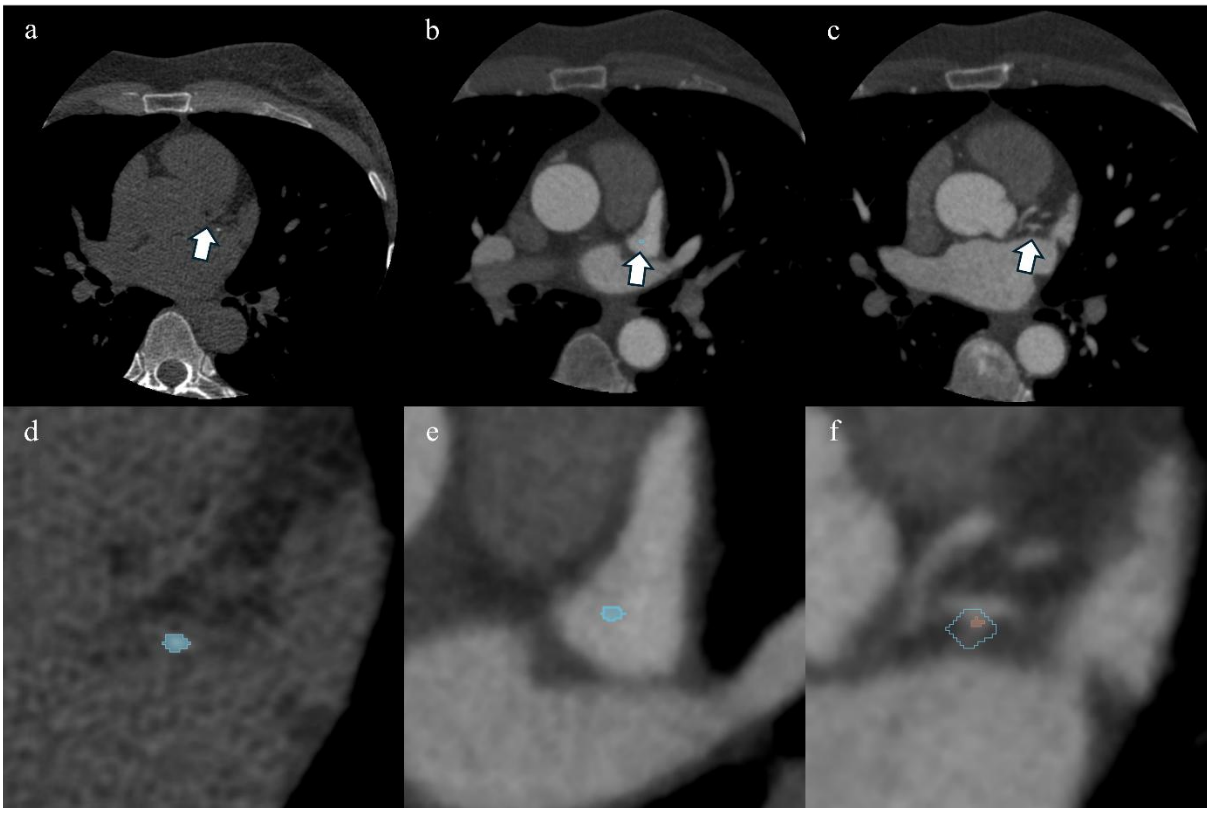

Figure 1: Semi-automatic multimodal plaque quantification with coronary CT angiography (CCTA) (**b**, **c**, **e**, **f**) and registered thin-slice non-contrast CT (NCCT) (**a**, **d**). Calcified plaque (blue shape) missed by CCTA (white arrow in **a**, **c**) is misaligned before registration (**b**, **e**). After registration, and dilation (see Methods) calcified plaque overlays (blue outline, **f**) with plaque missed by CCTA (red, **f**).

Table 2: Elastix [1] parameters to reproduce the two-stage registration framework. Optimization was performed on an Intel Core i9-10900X @ 3.70 GHz and 32 GB RAM and takes 3-5 minutes per 3D volume (512x512 matrix) depending on the number of slices. No dedicated GPU is required, the optimization and registration workflow runs entirely on the CPU. Advanced Mattes Mutual Information (AMM), Advanced Normalized Correlation (ANC), Adaptive Stochastic Gradient Descent (ASGD).

	T_1 (x)		T_2 (x) (masked)
	Affine	B-spline (coarse)	B-spline (fine)
Iterations	200	500	500
Metric	AMM	ANC	AMM
Metric 2		Bending Energy	Bending Energy
Optimizer	ASGD	ASGD	ASGD
Pyramid Levels	3	5	5
Final Grid Spacing		10mm	5mm

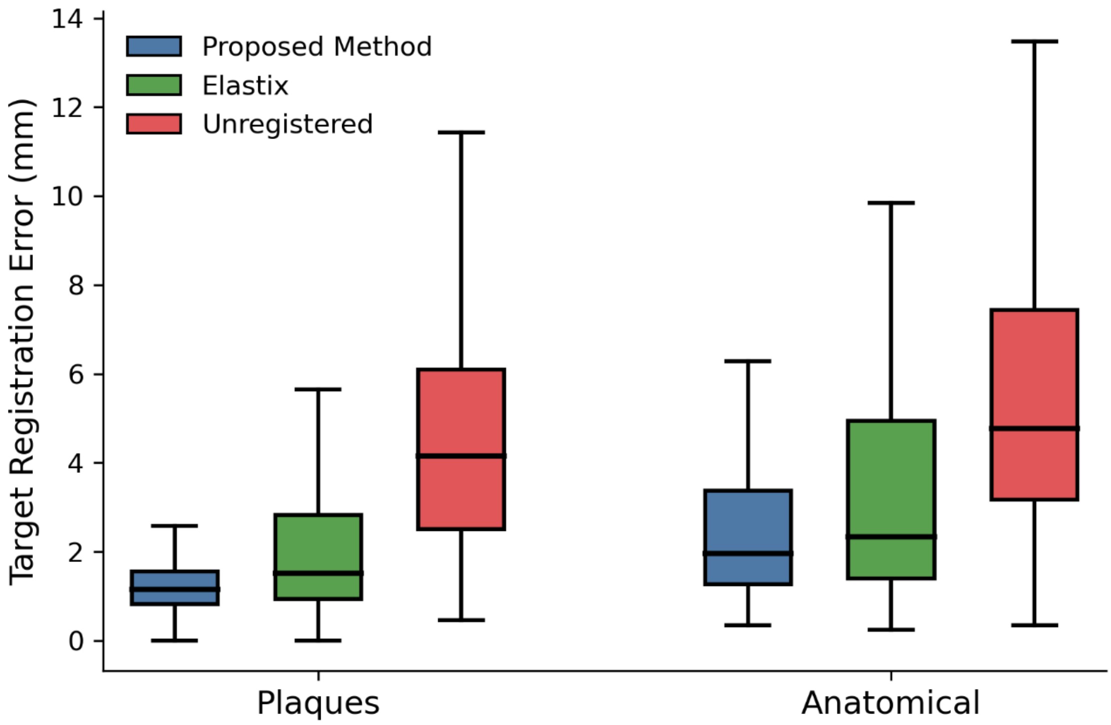

Figure 2: The proposed deep learning–aided registration method was compared with a cardiac registration approach from the Elastix Model Zoo (Par0044) [1]. Registration performance was assessed using the target registration error (TRE), calculated from up to four corresponding plaque-based and anatomical landmarks placed on each of the thin-slice non-contrast and contrast-enhanced CT images (n=45). Anatomical landmarks were defined at coronary branch points or other clearly identifiable orientation structures, with one landmark placed in each of the right coronary artery (RCA), left main (LM), left anterior descending (LAD), and left circumflex (LCX) arteries.

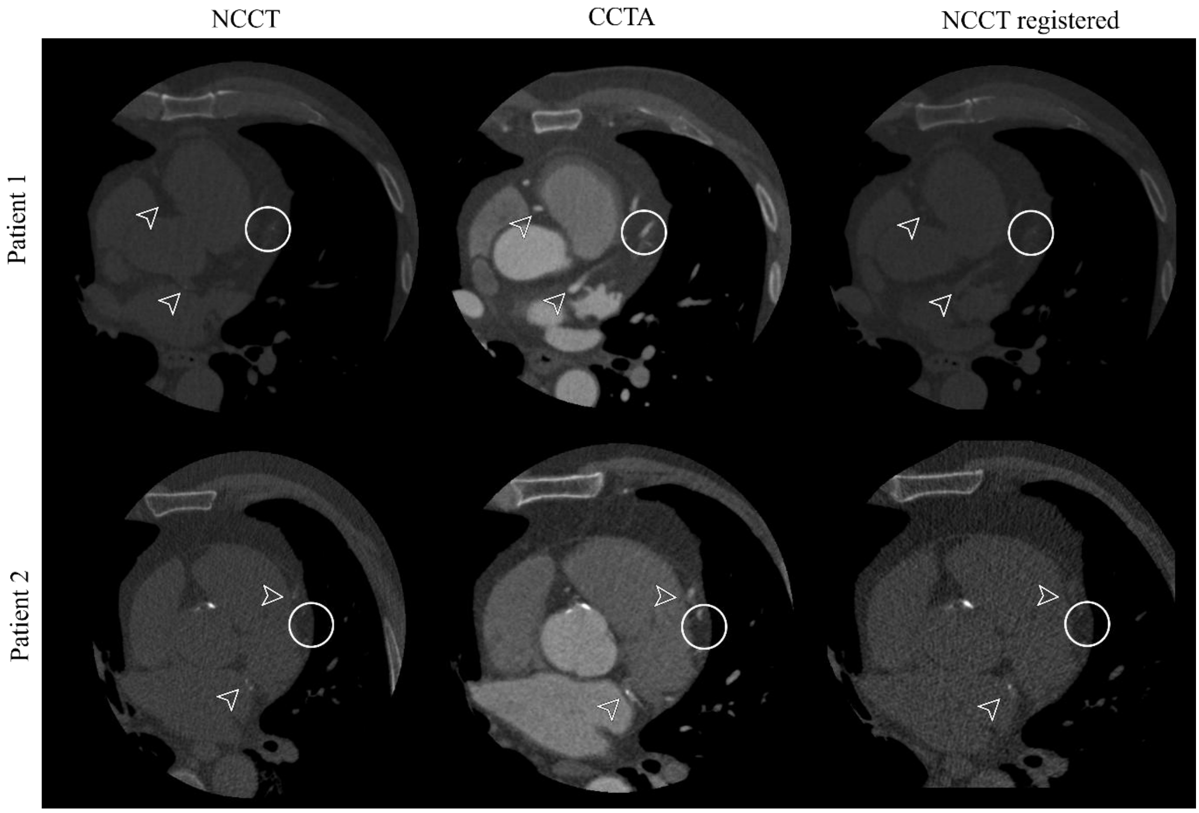

Figure 3: Illustration of the image registration process between non-contrast CT and CT angiography. For illustrative purposes, these figures have been initially aligned on the z-axis, with circles denoting a chosen common reference point visible in both modalities. Arrows point to specific regions where precise alignment is necessary for the registration algorithm.

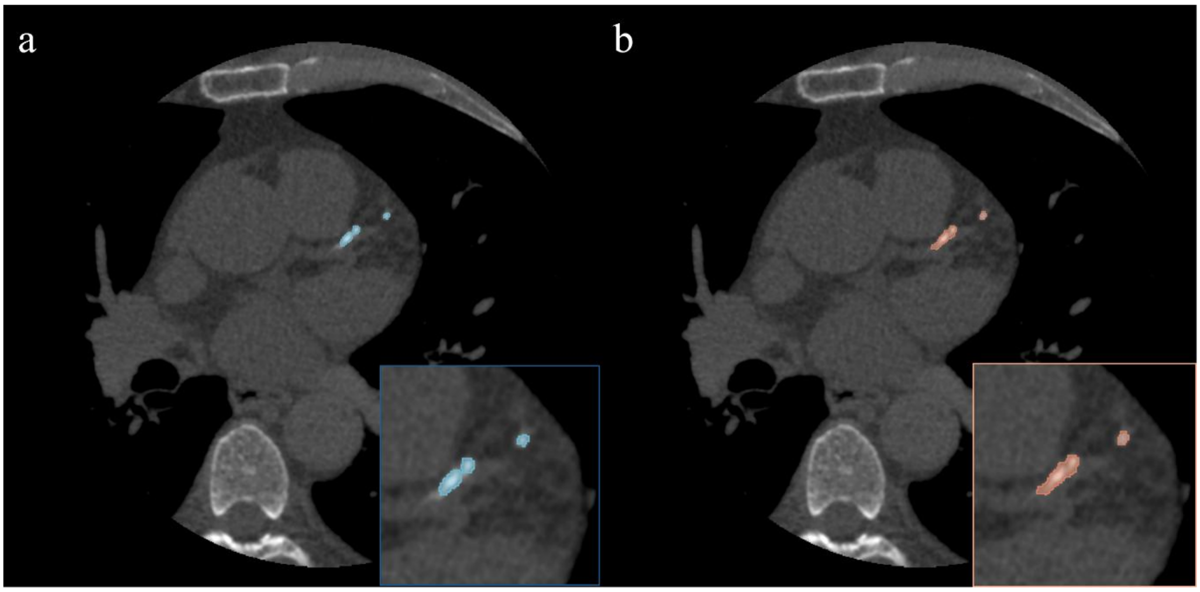

Figure 4: Comparison of both non-contrast CT observers (**a**, **b**). While both observers differed in the amount of segmented calcified plaque in terms of volume the number of plaques is consistent.

References

1. Klein, S., M. Staring, K. Murphy, M.A. Viergever, and J.P. Pluim (2010) elastix: a toolbox for intensity-based medical image registration. IEEE Trans Med Imaging 29:196-205.
